# Nucleocytoplasmic Shuttling of Influenza A Virus Proteins

**DOI:** 10.3390/v7052668

**Published:** 2015-05-22

**Authors:** Jing Li, Meng Yu, Weinan Zheng, Wenjun Liu

**Affiliations:** 1 Key Laboratory of Pathogenic Microbiology and Immunology, Institute of Microbiology, Chinese Academy of Sciences, Beijing 100101, China; E-Mails: lj418@163.com (J.L.); zhengdayumeng@163.com (M.Y.); zwn@foxmail.com (W.Z.); 2 University of Chinese Academy of Sciences, Beijing 100049, China

**Keywords:** influenza A virus, nucleocytoplasmic shuttling, virus replication

## Abstract

Influenza viruses transcribe and replicate their genomes in the nuclei of infected host cells. The viral ribonucleoprotein (vRNP) complex of influenza virus is the essential genetic unit of the virus. The viral proteins play important roles in multiple processes, including virus structural maintenance, mediating nucleocytoplasmic shuttling of the vRNP complex, virus particle assembly, and budding. Nucleocytoplasmic shuttling of viral proteins occurs throughout the entire virus life cycle. This review mainly focuses on matrix protein (M1), nucleoprotein (NP), nonstructural protein (NS1), and nuclear export protein (NEP), summarizing the mechanisms of their nucleocytoplasmic shuttling and the regulation of virus replication through their phosphorylation to further understand the regulation of nucleocytoplasmic shuttling in host adaptation of the viruses.

## 1. Introduction

Influenza A virus (IAV), which contains an eight-segment, single-stranded, negative-sense RNA genome, is a member of the Orthomyxovirus family. Further subtyping is based on the antigenicity of the hemagglutinin (HA) and neuramindase (NA) surface glycoproteins. Wild waterfowl are the natural reservoir of IAV, which can infect many kinds of mammals, including humans [1]. Currently, two major subtype (H1N1 and H3N2) viruses are circulating among humans. Although still an area of active research, the prevention and control of IAV infection mainly rely on vaccines, and the trivalent inactivated vaccine, which is recommended by the World Health Organization (WHO), is widely used.

## 2. Influenza A Virus Proteins

Influenza virions are roughly spherical, made up of a viral envelope, a matrix layer, and a central core. The envelope consists of HA and NA, usually in a 4:1 ratio [2], and the M2 protein acts as a proton channel. The oligomerization of M1 forms the matrix layer to stabilize the structure of the virion [3,4]. The trimeric polymerase complex (composed of PB1, PB2, and PA), nucleoprotein (NP), and viral RNA constitute the basic unit of the IAV genome for transcription and replication, which is called the viral RNA ribonucleoprotein (vRNP) [5]. In addition, IAV also encodes several other proteins, such as nuclear export protein (NEP), which participates in export of newly made vRNPs; nonstructural protein 1 (NS1), which mainly inhibits the type I interferon (IFN-I) system of host cells; and PB1-F2, PB1-N40, and PA-X, PA-N155, PA-N182, M42, and NS3 [6,7,8,9,10,11], which all play various roles in the virus life cycle.

Unlike most RNA viruses, IAV replicates in the host nucleus [12]. In the early stage of infection, vRNPs must enter the nucleus for replication and transcription. Then, the newly synthesized proteins translocate back into the nucleus to help in the assembly and nuclear export of progeny vRNPs. Finally, the formation of mature virions and budding occurs. Thus, nucleocytoplasmic shuttling is important throughout the entire IAV life cycle. The transport of proteins, as well as cellular and viral RNPs, from the cytoplasm to the nucleus (and *vice versa*) is an active, energy-dependent, signal-mediated process. Taking into account that virus RNAs transport as RNPs rather than naked RNAs, RNA-binding proteins play a key role in RNA transport. Most of these proteins both bind RNA and contain sequences that allow them to be exported/imported between the cytoplasm and nucleus. In this review, we discuss key findings that have advanced our understanding of the nucleocytoplasmic shuttling of influenza virus proteins (especially for the M1, NP, and NS protein) and their influence on virus replication.

## 3. Nucleocytoplasmic Shuttling Mechanism of IAV Proteins

In eukaryotic cells, the transport of proteins and RNAs into and out of the nucleus through nuclear pore complexes (NPCs)-large macromolecular structures embedded in the double membrane of the nuclear envelope, requires the help of transporter molecules and is an active process. There are two types of transporter molecules, named importin and exportin based on their functions. The importins, α and β, both belong to the importin β super-family. The former is able to recognize and bind the nuclear location signals (NLSs) in the target protein, and the complex is recognized and bound by importin β, which subsequently interacts with the NPC to accomplish the translocation of target proteins. Like the importins, exportins are responsible for ferrying cargo out of the nucleus via the same working principle. Chromosome region maintenance protein 1 (CRM1) is an exportin that recognizes and binds to target proteins carrying a nuclear export signal (NES) and mediates their export from the nucleus [13]. The classic NLSs recognized by importin α consist of two classes. The first is represented by the NLS of the Simian virus 40 (SV40) Large T-antigen (LT) protein and is composed of several contiguous basic amino acids (^126^P**KKKRK**V^132^, the basic amino acids are shown in bold). The second category is represented by the NLS of nucleoplasmin, which is characterized by continuous basic amino acid sequences at both ends separated by >10 random amino acid residues (^156^**KR**PAATKKAGQA**KKKK**^171^, the basic amino acids are shown in bold) [14,15,16]. There are many subtypes in the importin α family, with different cargo specificities for different NLSs. Additionally, the conformation and phosphorylation of target proteins’ NLSs are also associated with importin α [17]. The NES recognized by CRM1 is composed of hydrophobic amino acids with larger side chains that appear at regular intervals. Its characteristic sequence is Φ-X(2-3)-Φ-X(2-3)-Φ-X-Φ, where Φ stands for Leu, Val, Ile, Met, or Phe, and X can be any amino acid [18].

After the first NLS of SV40 LT was identified in 1984, functional NLSs and NESs were subsequently found in influenza virus proteins, including PB1, PB2, PA, NP, M1, NS1, and NEP (Table 1). The proteins with NLSs can be recognized and bound by importin α for nuclear entry. Another non-classical nuclear import factor, Ran binding protein 5 (RanBP5), interacts with PB1-PA dimer to transport into the nucleus [19,20]. After the completion of replication, vRNPs require the help of two viral proteins, M1 and NEP, which contain the NESs for recognition by CRM1 to move out of the nucleus for assembly and budding of progeny viruses [21,22,23,24]. The following section describes the nucleocytoplasmic shuttling mechanism of four IAV proteins (M1, NP, NS1, and NEP) in detail.

### 3.1. Nucleocytoplasmic Shuttling of M1

**Figure 1:**
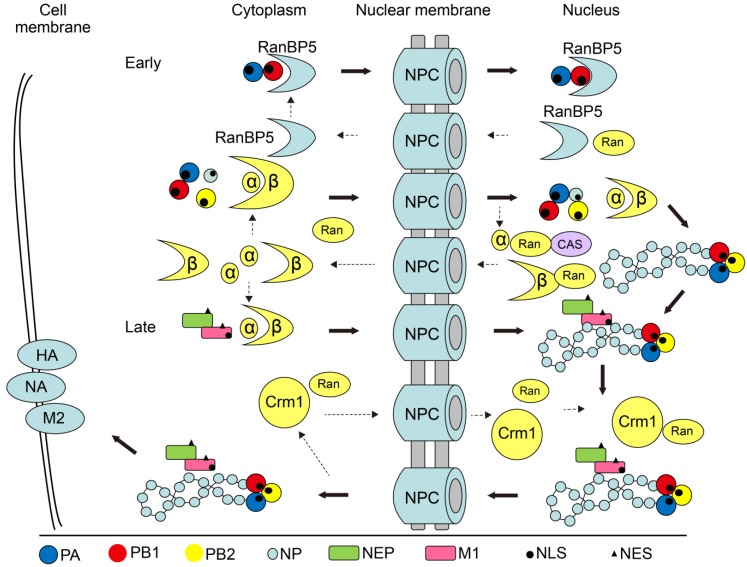
Model of the nucleocytoplasmic shuttling of influenza virus proteins and vRNPs. The importin/exportin molecules recognize and bind the NLSs/NESs in the target proteins and then are bound by the importin/exportin molecule, which subsequently interacts with the NPC to accomplish the nucleocytoplasmic shuttling. The importins α-β and RanBP5 pathway separately involved into the vRNPs import. CRM1 is a commonly utilized exportin for influenza virus vRNPs. The export of IAV RNPs seems to depend on a “RanGTP-CRM1-NEP-M1-RNP” model for transport out of the nucleus as a complex.

M1 is the most abundant protein in the virion and forms the matrix layer inside the virus envelope. The exterior matrix layer combines with HA, NA, and M2 located on the envelope, and the interior matrix layer combines with vRNP. Both layers form the virion structure. Initially, it was thought that M1 interacted with HA and NA inside the cytoplasm. Subsequently, the M1 protein was found to enter the nucleus and be indispensable for vRNP nuclear export [25]. Aside from the well-characterized basic amino acid-rich NLS [26], there is another basic amino acid stretch at position 76–78 that is highly conserved among influenza A and B virus M1 proteins and plays a critical role in virus replication. Indeed, there is evidence showing that R77A or R78A substitutions in M1 result in aberrant M1 intracellular localization and defects in virus assembly and budding [27]. The viral polymerase proteins (PA, PB1, and PB2) are imported into the nucleus to assemble vRNPs, together with NP and viral RNA. It is well established that vRNP nuclear export is mediated by the nuclear presence of M1 and NEP. In the absence of a functional NES, M1 is not able to interact with CRM1 itself. NEP likely bridges the M1 and CRM1 interaction by binding to M1 with its C-terminus, while the two N-terminal NESs interact with CRM1 and allow access to the CRM1-dependent export pathway (Figure 1). Our pervious experiments show that intracellular distribution of M1 is not sensitive to the CRM1 inhibitor leptomycin B (LMB), further confirming the indispensable role of NEP for vRNP nuclear export. Furthermore, the M1 individual nuclear export was specially dependent on its NES and critical for influenza A virus replication [24].

**Table 1:** List of influenza A virus protein nuclear localization signals (NLSs) and nuclear export signals (NESs).

Protein	NLS	NES	References
NS1	NLS1: ^34^RLRR^38^, highly conserved	^138^FDRLETLILL^147^	[28]
	NLS2: ^216^PKQKRK^221^, two NLSs act independently		[29]
PB1	^187^RKRRVRDNMTKKMVTQRTIGKRKQR^211^, bipartite NLS		[30] [31]
PB2	NLS1:^449^GIESIDNVMGMIGILPDMTPSTEMSMRGVRISKMGVDETSSAEKIV^495^, required for efficient import NLS2: ^736^KRKR^739^, bipartite NLS K736 required for efficient import		[32] [33]
PA	NLS1: ^124^RREVHIYYLEKANKIK^139^, bipartite NLS NLS2: 186–247 E154 required for efficient import		[34]
M1	^101^RKLKR^105^	^59^ILGFVFTLTV^68^ L66A, V68A mutation impairs M1 nuclear export	[26] [24]
NP	NLS1: ^3^TKGTKRSYEQM^13^, unconventional NLS, 3Ser crucial for N-terminal phosphorylation NLS2:^198^KGINDRNFWRGENGRRTR^216^, bipartite NLS	NES1:^24^EIRASVGKMIDGIGRFYIQMCTELKL^49^ NES2: ^183^VKGVGTMVMELIRMI^197^ NES3:^248^PGNAEFEDLIFLARSALILRGSVAHKS^274^	[35] [36] [37] [38] [39] [40] [41] [42]
NEP	Passive diffusion, no need of NLS	NES1: ^12^ILMRMSKMQL^21^ NES2: ^31^IITQFESLKI^40^	[21] [43] [44]

### 3.2. Nucleocytoplasmic Shuttling of NP

There are at least two NLSs in NP, including the unconventional NLS1 (3–13), which interacts with importin α [36], and NLS2 (198–216) [39], which together with NLS1 was originally hypothesized to be a traditional bipartite NLS. However, the crystal structure of NP shows that the two clusters are too close to be recognized by importins as a bipartite NLS [45]. Although it is hard to judge which of the two NLSs contributes most to the import of vRNP, import is almost completely inhibited when both NLSs are blocked using antibodies [46,47]. In addition, *in vitro* experiments and a reverse genetics approach reveal that NLS1 plays a pivotal role in mediating vRNP nuclear import [48,49].

Three NESs have been identified in NP that have the familiar sequence characteristics of common export signals. Two of them do not rely on CRM1 for export, though the third does. Further, all three must be simultaneously mutated to inhibit NP nuclear export, proving their functional independence [42]. The three NESs are closely related to the nuclear export of vRNP, and mutation of the NP NESs inhibits the replication of the virus in the post-transcriptional stage. Through function comparison via mutation of all seven NESs in IAV proteins, it was found that NP NES3 played a key role in the replication of the virus, but the mechanism for this phenomenon has not been elucidated [50].

### 3.3. Nucleocytoplasmic Shuttling of NS1

NS1 is mainly located inside the nucleus and transports to the cytoplasm in the late stage of infection. Two NLSs and one NES have been identified in NS1 [28]. S221 in NLS2 is very important for the nuclear localization of NS1. Further, the nuclear export of NS1 does not (or only partially) rely on CRM1 [51]. By comparison among different strains, it was found that the NS1 NES is highly conserved, and mutations of conserved residues, such as L141A and A149V, lead to the retention of NS1 inside the nucleus during the late stage of infection [52].

### 3.4. Nucleocytoplasmic Shuttling of NEP

NEP is known as a nuclear export protein. It may not need a NLS as it is only 14 kDa in size, and at present, no NLS has been found in NEP. Through the use of an inhibitor of NLS-dependent active nuclear transport and an energy depletion assay, it was demonstrated that NEP enters the nucleus by passive diffusion [44].

NEP is involved in the nuclear export of vRNPs [53]. NEP is able to interact with CRM1 in mammalian two-hybrid systems, suggesting that the nuclear export of NEP depends on binding to CRM1 [54]. Further, it has been demonstrated that the NES1 and NES2 of NEP [43] are CRM1 dependent and jointly participate in the nuclear export of NEP and vRNP. Interestingly, the NEPs of different strains result in distinct cellular distribution patterns, forming unique nuclear aggregates, with the weaker binding capacity to CRM1 due to one amino acid change in the NES, perhaps facilitating the export of the vRNP complex [44]. 

## 4. Biological Significance of Nucleocytoplasmic Shuttling on IAV Replication

### 4.1. Interactions among Members of the vRNP Nucleocytoplasmic Shuttling Complex

The vRNP is the basic unit for the replication and transcription of IAV. The interaction between members of the vRNP complex is of great significance for the structural stability and function of the complex. Several functional domains of related proteins involved in the interaction with the members of the polymerase complex [55,56]. For example, several binding sites on NP have been identified, including two NP-NP interaction regions (aa 189–358 and 371–469), two RNA interaction regions (aa 1–77 and 79–180), two PB2 interaction regions (aa 1–166 and 255–498), and three polymerase interaction regions (R204, W207, and R208) [57,58]. As with the relevant interaction regions in the trimeric polymerase, these regions support the formation of the vRNP and are required for its function. It has been found that disturbing the self-polymerization of NP and destroying the interaction between NP and PB2 or NP and PB1 regulate the replication of influenza virus in a negative way [59]. The C-terminus of NEP also interacts with PB2 and PB1 in the viral polymerase complex to regulate the activity of the polymerase, resulting in alteration of the synthesis of vRNA [60].

In the process of infection, IAV enters the host cytoplasm, and at the same time, the M2 ion channel opens, internally acidifying the virus. Subsequently, the matrix layer depolymerizes, and as a result, M1 and vRNP dissociate from each other [55]. This conformational change results in the exposure of the NLS, which leads to the entry of the vRNP into the nucleus for replication and transcription. Each polymerase subunit and NP contain their own NLSs, and it is possible that the monomers are transported into the nucleus (for NP and PB2) or that PB1-PA dimers or trimers form in the cytoplasm before nuclear import, perhaps as part of a mechanism regulating different phases of the infection [61]. It has been intriguing trying to identify which NLSs play a decisive role in transport into the nucleus. Indeed, structure-function experiments demonstrate that the role of NLS1 is very important in mediating vRNP nuclear localization [62].

For vRNP nuclear export, a “CRM1-NEP-M1-vRNP” complex forms, where CRM1 recognizes and binds to the NES on NEP, accompanied by GTP hydrolysis (Figure 1). Thus, the nuclear export of vRNP depends on CRM1 [63]. Although NP contains an NES and can interact with CRM1, the NP/CRM1 interaction itself fails to induce the hydrolysis of RanGTP, suggesting that the NESs on NP cannot directly mediate vRNP nuclear export [23]. Indeed, M1 and NEP are necessary for this process [21,25]. M1 is exported from the nucleus via a CRM1-independent pathway, while all of the NESs of NEP are CRM1 dependent, suggesting that the NES on NEP directly mediates the nuclear export of vRNP. However, NES mutation on M1 leads to the nuclear retention of M1 and the accumulation of NEP, in addition to significantly reducing the virus titer, indicating that M1 plays an important role in vRNP nuclear export [24]. When the vRNP translocates out of the nucleus, the C-terminus of NEP binds to the N-terminus of M1, covering the NLS on M1 and inhibiting vRNP re-entry into the nucleus; mutation of NEP W78 destroys the interaction between NEP and M1 [23]. Experiments also suggest that the ability of NEP to support the nuclear export of vRNPs by stabilizing the interaction between M1 and vRNPs requires the NES-containing N-terminus, as well as the last three amino acids of NEP, and is functionally linked to the polymerase activity-enhancing function of NEP during viral genome replication [64].

### 4.2. Phosphorylation of IAV Proteins to Regulate Nucleocytoplasmic Shuttling

**Figure 2:**
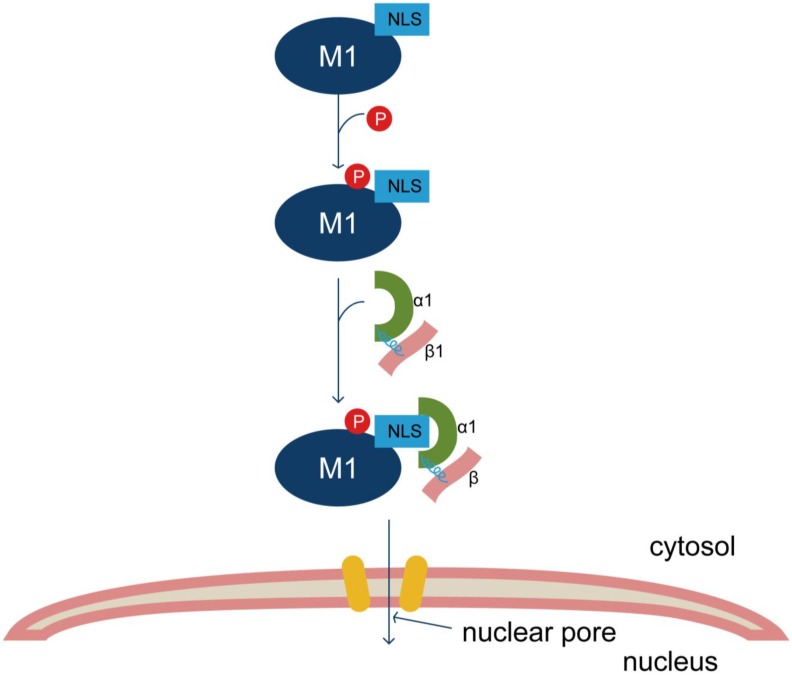
Model of Y132 phosphorylation participating in nuclear import of IAV M1. The conserved tyrosine 132 of M1 is a phosphorylation site. The NLS-neighboring phosphorylated Y132 residue is crucial for the cytoplasmic-nuclear translocation of M1 by increasing the binding capacity of M1 and nuclear import factor importin-α1, subsequently affecting the replication of IAV.

The phosphorylation of IAV proteins is an important regulatory mechanism that influences viral replication by affecting the nucleocytoplasmic shuttling of both the individual viral proteins themselves and the vRNP complex. Nuclear transport of M1 depends on phosphorylation mediated by protein kinase C (PKC), as PKC inhibition effectively decreases virus replication [65]. Mutation of the M1 Y132 phosphorylation site in A/WSN/1933 (H1N1) eliminates the interaction between M1 and importin α1, inhibiting M1 nuclear import, and thus having a negative influence on virus replication. JAK kinase inhibition can block this phosphorylation and impair WSN M1 entry into the nucleus but not H9N2 M1, suggesting that differential regulation of the interaction between M1 and importins exists in different subtypes of IAVs [66] (Figure 2). There are additional potential phosphorylation sites in the IAV M1 protein, such as T108, that are close to the NLS but whose functions have not been determined [67].

NP is rich in serine residues, which is crucial for its phosphorylation, especially on the N-terminus of NLS1 [37]. Inhibiting phosphorylation of infected cells stabilizes the localization of NP in the nucleus and inhibits its nuclear export [38,68]. Several highly conserved phosphorylation sites have been identified in NP, such as S9/Y10 and S165. Mutation of the former significantly decreases viral titers [67], and the S165D mutation (which mimics phosphorylation) may maintain NP in a monomeric state, reducing its affinity for RNA [69]. In our lab, we demonstrated that the phosphorylation of S9, Y10, and Y296 was essential for virus growth in cell culture and modulation of activity of the viral polymerase in a mouse model and the nuclear-cytoplasmic shuttling of NP. The phosphorylation and de-phosphorylation of S9 and Y10 controlled nuclear import of NP by affecting the binding affinity between NP and importin-α. In addition, the phosphorylation of Y296 caused nuclear retention of NP by reducing the interaction between NP and CRM1 [70].

The phosphorylation of T215 in NS1 is mediated by the CDK/ERK pathway, and the T215A mutation attenuates virus replication [71]. The functional analysis of several phosphorylation sites, including T215, S48, and S42, shows that the S42D mutation eliminates dsRNA binding by NS1, also attenuating virus replication [72].

## 5. Conclusions

To adapt to new host species, influenza viruses must overcome significant barriers to cross-species transmission and replication. The changes in the receptor-binding properties of the viral HA to adapt to new host species are well characterized, but the nuclear import machinery to determine the host tropism requires a better understanding. Previous studies show that the preference of PB2 for different importin-α isoforms (α1, α3, α4, α5, α6, and α7) differs between hosts. For efficient virus replication in different hosts, all viruses depend on importin-α1. Meanwhile, the polymerase subunit PB2 of avian viruses requires importin-α3, PB2 of mammalian virus shows importin-α7 specificity, and H1N1v replication depends on both, suggesting ongoing adaptation of this virus [73]. PB2 specificity for given importin-α isoform is mediated by both NLS and protein context. Researchers have found out PB2 627-NLS domain can induce selective importin binding in host-specific manner and areas where more research is required, including other transport associated protein in IAV. The specific interaction of the polymerase subunit PB2 and the NP of mammalian viruses with importin-α3/7 regulates the efficacy of viral replication and determine host range underlining the importance of the nuclear envelope in interspecies transmission [73,74,75]. Work in our laboratory also demonstrates differences in the nucleocytoplasmic shuttling of NPs between human-origin WSN and avian-origin IAVs, which impact the replication, adaptation, and pathogenicity of the viruses (unpublished). In addition, we have also found that the CA04 strain displays different vRNP distribution patterns, forming unique nuclear aggregates, with respect to WSN by comparing different strains of H1N1 NEP for vRNP nuclear translocation. In the further study, we confirmed that CA04 NEP interacts less efficiently with CRM1 and site T48 is responsible for the nuclear aggregation [44]. In view of NEP playing an important role in viral polymerase activity and vRNP nuclear transport efficiency, these differences may be one reason for divergence of the pathogenicity and host adaptation range between these virus strains. Future work will dissect the finely tuned interactions induced by natural/pressure selection to acquire cross-species activity.

Phosphorylation is one of the most common and efficient post-translational modification methods in organisms, playing an important role in regulating many metabolic pathways. Nucleocytoplasmic shuttling of proteins and phosphorylation are closely related. Phosphorylation and dephosphorylation can strengthen or weaken the nucleocytoplasmic shuttling of a protein through a variety of mechanisms. For instance, phosphorylation of key NLS or NES residues on viral proteins can strengthen the affinity between the proteins and their nuclear transport receptors [76]. Alternatively, phosphorylation can strengthen the ability of a protein to separate itself from the NPC. In addition, phosphorylation can lead to a conformational change in the protein, selectively exposing its NLS or NES. In contrast, phosphorylation of the NPC or virus proteins can also inhibit nucleocytoplasmic shuttling. Regardless, research into the mechanism of how phosphorylation regulates nucleocytoplasmic shuttling is still at an early stage. Development of better quantitative methods to determine the influence of phosphorylation on the dynamic processes of protein transport will lead to a more comprehensive understanding of the role of phosphorylation in the IAV life cycle, which is beneficial for influenza prevention and control. In addition, sumoylation is critical for protein-protein interactions in many biological systems. For example, the sumoylation of matrix protein M1 modulate the assembly and morphogenesis of influenza A virus [77,78], or sumoylation of NP is indispensable for virus growth and intracellular trafficking [79]. Further research found out that the sumoylation of M1 promotes M1-mediated vRNP nuclear export to increase viral replication [80]. Thereby, the modification of virus/cellular protein to further regulate the virus life cycle needs to be given more attention for further research.
